# The Need for Health Care Innovation Training in Medical Education

**DOI:** 10.2196/79489

**Published:** 2025-12-19

**Authors:** Lily Zhu, Jeffrey Khong, Oren Wei, Katherine C Chretien, Youseph Yazdi

**Affiliations:** 1 School of Medicine, Johns Hopkins University, 733 N Broadway, Baltimore, MD, 21205, United States; 2 Department of Biomedical Engineering, Johns Hopkins University, Baltimore, MD, United States

**Keywords:** health care innovation, medical education, curriculum development, bioengineering, leadership

## Abstract

The rapid transformation of the health care landscape requires physicians to not only be skilled clinically but also navigate and lead a highly dynamic, innovation-driven environment. This also provides an avenue for physicians to significantly enhance their ability to help their patients, through participation in health innovation projects. Despite this growing need and opportunity, few medical schools provide formal training in innovation and entrepreneurship (I&E). In this perspective, we examine the need for I&E education in medical curricula by exploring student interest, effective program models, and implementation strategies. To better understand medical student interest in innovation and willingness to participate in I&E programs during medical school, we surveyed 480 medical students at our institution, the Johns Hopkins University School of Medicine, and received 90 responses with a 19% response rate. We observed a strong interest in health care I&E, with 97% (87/90) of respondents valuing knowledge or experience in I&E and 63% (56/90) expressing intent to incorporate I&E into their careers. To assess the real-world impact of I&E education on medical professionals, we surveyed 12 alumni of the Johns Hopkins Center for Bioengineering Innovation and Design (CBID) Master’s program who had also completed medical school. Graduates reported that their experiences cultivated transferable skills—design thinking, interdisciplinary collaboration, and leadership—that shaped their professional trajectories. We propose three models for incorporating I&E education into existing medical curricula—short-term workshops, one-year gap programs, and longitudinal tracks—and discuss their advantages and trade-offs. Early and structured exposure to I&E education in medical school empowers students to identify unmet clinical needs, collaborate across disciplines, and develop real-world solutions. As the pace of innovation continues to accelerate, integration of I&E education into medical curricula offers a timely opportunity for medical schools to cultivate physician leaders in this space.

## Introduction

Medical students traditionally receive extensive training in biological sciences, clinical reasoning, and patient care, but minimal exposure to innovation methodologies, design thinking, and entrepreneurial concepts [1]. As the health care landscape undergoes unprecedented transformation through technological advances, changing information and data landscapes, and evolving patient expectations, traditional medical school curricula that primarily emphasize disease diagnosis and treatment protocols may no longer fully prepare physicians for the opportunities and challenges they will encounter [2,3]. As medical students, educators, and administrators, we believe that health care innovation and entrepreneurship (I&E) represents a critical area of education that should be integrated into modern medical school curricula. These concepts provide an important framework for how to identify real-world health care challenges, characterize these challenges in detail, and design effective solutions. They mix both theory and practice, spanning four essential domains: Medical, Business, Technical, and Entrepreneurial. From a medical student perspective, we uniquely recognize the critical gap between the rapidly evolving health care environment we are entering and the limited exposure to I&E concepts during medical education. Physicians are uniquely positioned to identify clinical problems worth solving, yet most lack the formal training to translate these insights into viable solutions, representing a missed opportunity to significantly expand their impact on the care of patients around the world.

## Current Landscape and Needs

Although there has been increasing recognition of the importance of health care innovation, few medical schools offer structured I&E programs [4]. Examples include Harvard’s Health Sciences and Technology (HST) program, Stanford’s Biodesign Innovation coursework for medical students, University of Michigan’s Biomedical Innovation & Entrepreneurship Certificate Program, George Washington’s Clinical Practice Innovation and Entrepreneurship Track, and at Johns Hopkins, the Center for Bioengineering Innovation & Design (CBID)’s one-year full time Master’s program. Since its inception in 2009, 24 medical students and physicians have completed the CBID program [5].

A 2021 study found that only 15.2% (26/171) of American and Canadian allopathic medical schools offered I&E-oriented medical education programs [6]. Among these 28 programs, 57% (n=16) had been started within the past four years, 75% (n=21) required a selective application process, and 79% (n=22) required students to complete a capstone project [6]. The most common program structure is a four-year track or concentration (15/28, 54%) [6]. Other formats vary in duration, ranging from weeks-long courses to a five-year dual-degree program. Teaching strategies in these I&E programs include lecture series, progress meetings, problem-based learning, workshops, mentorship, and guest lectures.

## Survey of Student Interest

Given this growing but still limited landscape of opportunities, we sought to assess medical student interest in health care I&E programs at our institution, the Johns Hopkins University School of Medicine. During the 2023‐2024 academic year, we distributed an electronic survey via email to 480 students across all years and received 90 responses, an approximately 19% response rate. The survey assessed student perceptions of innovation importance and career intentions and was approved by the Johns Hopkins Medicine Institutional Review Board (ID: IRB00381640).

The results suggest strong interest among the student body in integrating health care I&E into medical education and a recognition of its importance. Most respondents (n=87, 97%) expressed that a physician’s knowledge and experience with health care innovation was at least somewhat important, including 39 students (44.8%) who thought it was very important or essential. Additionally, 63% of students expressed that they were likely or very likely to incorporate health care innovation into their future practice. A total of 36% percent of students indicated that they were likely to devote more than one quarter of their future career time to health care innovation. Importantly, 43% of students reported that the availability of opportunities to learn about innovation was a positive factor in their choice of medical school (Table 1).

**Table 1.** Medical student survey.

Survey questions	Respondents, n (%)
How important is it for a physician to have knowledge about and experience with health care innovation?	
Not important	3 (3)
Somewhat important	48 (54)
Very important	37 (41)
Essential	2 (2)
Thinking about your vision for your medical career long term, what percentage of your time do you anticipate devoting to health care innovation?	
0%‐25%	58 (64)
25%‐50%	26 (29)
50%‐75%	4 (5)
75%‐100%	2 (2)
When you were applying to medical school, how did the availability of opportunities for exposure to healthcare innovation affect your choice of medical school?	
It was a negative factor	0 (0)
It was not a factor	51 (57)
It was a minor positive factor	21 (23)
It was a moderate positive factor	13 (14)
It was a major positive factor	5 (6)

We acknowledge several limitations of our student survey. The relatively low response rate may introduce selection bias, as students with a preexisting interest in I&E may have been more motivated to respond. Second, the survey reflects the perspectives of students at a single institution, and these findings may not be generalizable to other institutions. Nevertheless, with nearly all respondents considering familiarity with I&E somewhat important, this represents a sizable number of students, even factoring in the response rate.

## Impact of Health Care Innovation and Entrepreneurship Training

Our institution offers a one-year immersive Master’s degree program managed by CBID that provides a structured framework to allow students to study medical device innovation in a hands-on, team-based format, with attention to all four domains mentioned previously. To better understand the impact of early exposure to health care I&E, we distributed an electronic survey via email to 12 medical school graduates who completed the program between 2018 and 2024.

Five graduates had completed the CBID program during medical school, and seven after medical school. The respondents’ subsequent career paths demonstrate a strong commitment to I&E. Two practicing physicians currently dedicate 90% of their time to clinical practice and 10% to entrepreneurship, while ten current residents anticipate distributing their time across clinical practice (54%), research (19%), industry (9%), entrepreneurship (16%), and other roles (2%), including teaching programs like CBID (Table 2).

We also sought to assess the impact of skills that alumni had developed through the CBID program. When asked about which skills have been most applicable to their medical career, the most common responses were design thinking and ideation (n=11, 91.7%), leadership and team management (n=8, 67.7%), knowledge of health care markets (n=7, 58.3%), and interdisciplinary collaboration (n=6, 50%) (Table 2).

**Table 2.** CBID graduate survey.

Survey questions	Respondents
When did you complete the CBID program? n (%)	
During medical school	5 (42)
After medical school	7 (58)
How do you currently allocate your time to the following? n=2, (% of time)	
Clinical practice	90
Entrepreneurship	10
How do you hope to allocate your time to the following in your future career? n=10, (% of time)	
Clinical practice	54
Research	19
Entrepreneurship	16
Industry	9
Other	2
Which skills or experiences gained from CBID have been most applicable in your medical career? n=12, n (%)	
Design thinking and ideation	11 (92)
Leadership and team management	8 (68)
Knowledge of health care markets	7 (58)
Interdisciplinary collaboration	6 (50)
Project management	3 (25)
Other	1 (8)

a CBID: Center for Bioengineering Innovation and Design

Participants highlighted the program’s transformative impact, with one stating, “The full CBID experience made a big difference in my career trajectory and the exposure alone to the space was invaluable as this is something most medical students do not have access to and gives a unique perspective on health care.” Similarly, another student noted, “CBID offers a truly unique, collaborative, and hands-on experience that transformed my medical and surgical training. Participants learn to tackle clinical challenges comprehensively and without bias, enabling the development of clinically, commercially, and technically sound innovations.” These findings suggest that structured I&E education through programs like CBID can equip students with skills that complement existing clinical training and experiences that potentially shape students’ career trajectories.

In our opinion, design thinking skills acquired through such programs have the potential to augment a trainee’s development across multiple aspects of medical practice and professional growth. Design thinking entails an organized, iterative process in which one repeatedly interrogates user needs, defines aspects of a problem, and explores potential solutions to address those aspects. This framework trains students to approach clinical scenarios with a structured, problem-solving mindset which can improve diagnostic reasoning and clinical judgment [7-9]. Moreover, exposure to design thinking can empower clinicians to reimagine their career trajectories and even contribute to innovations such as the development of new medical devices and therapies [10,11].

Interestingly, while 50% (6/12) of alumni identified interdisciplinary collaboration as valuable, it ranked lower than expected given the inherently collaborative nature of health care. In clinical practice, physicians routinely coordinate with a wide range of professionals and specialists from other disciplines. Likewise, successful physician innovators depend on interdisciplinary teams including engineers, entrepreneurs, and business professionals to translate ideas into viable solutions. Navigating this ecosystem is a critical “soft skill” that I&E programs are uniquely positioned to cultivate through hands-on, team-based experiences. One possible explanation for these findings is that many medical students may have already developed a foundation of interdisciplinary skills by the time they joined the CBID program, which was applied to the innovation space with relative ease. Nonetheless, these findings do not detract the importance of supporting interdisciplinary collaboration through I&E curricula, as developing these skills early may better prepare future physicians to lead and thrive in complex health care environments.

Our alumni survey has limitations including a small sample size (n=12). Graduates of the CBID program are also likely to report that they plan to use this training in their future careers. However, our findings may actually underreport the true impact of such programs, as the long-term effects of such programs have not yet been fully realized. It may take time for graduates to assume leadership roles and make visible impacts in their respective fields.

## Critical Timing and Developmental Considerations

In our opinion, exposure to health care I&E is important early in medical education. When students are exposed to I&E training before clinical routines are developed, they are more likely to look for opportunities for improvement over existing practices and have the tools to act effectively on these opportunities. This timing allows students to see clinical situations and identify clinical challenges through a critical lens. Rather than being solely acclimated to current standards, these students are more likely to question assumptions, recognize unmet needs, and envision improved solutions. As current students ourselves who are deeply involved in I&E programs at Johns Hopkins, we can attest to the ways in which these opportunities have complemented our development of clinical knowledge and helped us better tackle patient care obstacles.

Additionally, we believe that early exposure to health care I&E can help to cultivate physician leaders in the health care innovation space. This is evident in the current and anticipated future professional activities of the CBID alumni. While many respondents are still in residency training, most remain actively involved in I&E through various pursuits. Seven graduates serve as chief executive officers or cofounders of startups, while others mentor engineering students (n=1) or lead research labs (n=1). These career trajectories suggest that early engagement with innovation during medical education not only enhances students’ ability to identify and solve complex clinical problems but also fosters leadership and sustained involvement in health care I&E.

## Structural Considerations

We believe that the goal of a medical school I&E program should be to expose students to the principles of design thinking, to provide them with practical experience navigating the design process, and to enable them to become conversant with a wide range of experts in this field. By the end of the program, students should be equipped with the skills necessary to apply the design process to real-world clinical problems. While we are aware that not all physicians will be interested in pursuing a career in health care I&E, these skills are applicable to all aspects of problem-solving in medical practice and will help students build sufficient fluency in order to collaborate with peers in related fields [8,9]. There are several models for incorporating health care I&E training into medical education, each with its own advantages and disadvantages (Table 3).

**Table 3.** Structural models for innovation and entrepreneurship programs.

Model	Duration	Advantages	Disadvantages
Short-term programs	Weeks to months	Broad accessibility Easy curriculum integration Low time commitment	Limited hands-on experience Theoretical focus
Gap year programs	1 year	Comprehensive immersion Complete project cycles Dedicated focus time	Financial barriers Limited accessibility
Longitudinal tracks	4 years	Integrated learning Extended project development	Challenges for sustained engagement Requires curriculum flexibility Resource intensive

## Short-Term Programs

Week- or month-long programs provide an excellent structure to introduce foundational I&E principles such as design thinking, problem identification, and prototyping to a broad range of students. Their shorter time commitment may be appealing to students who are interested but hesitant to dedicate significant time to I&E. Additionally, these programs are often easier to implement into the existing medical school curricula.

However, the limited duration may not provide students sufficient time for meaningful engagement in a hands-on project—an essential component for understanding the nuances of the design process. Without having practical experience, graduates of these curricula may only gain theoretical knowledge. Therefore, they may not be fully prepared to tackle and address more practical challenges that emerge along the journey of solving a clinical problem.

## Gap Year Programs (Eg, One-Year Master’s Degrees Such as the CBID Program)

Gap year programs provide comprehensive, hands-on experience working through the entire innovation process from identifying a clinical need to developing a prototype. By stepping away from traditional coursework and clerkships, students can fully immerse themselves in their projects without competing academic responsibilities, augmenting the learning experience.

However, financial or academic constraints may deter students from pursuing an additional year of education. Some students may be reluctant to delay their clinical training and career progression, while others interested in I&E may prefer earlier engagement rather than waiting several years to begin formal education.

## Longitudinal Tracks (Eg, Integrated Four-Year Programs Alongside the MD Curriculum)

A four-year longitudinal track can seamlessly integrate innovation-focused classes alongside traditional medical curriculum, allowing students to receive both theoretical and practical exposure in innovation. By introducing I&E training at the beginning of medical school, students have more time for hands-on exposure allowing for deeper engagement in the project, time for iterative problem-solving, and a greater chance of seeing projects through to implementation. Additionally, the extended time frame allows students to develop meaningful relationships with mentors in both academia and industry. This structure enriches the learning experience and helps prepare students with the skills needed to succeed as future innovators.

However, sustained engagement over four years can be difficult especially as competing academic demands or student interest change. Furthermore, not all medical school curricula allot the time and flexibility necessary to incorporate this coursework. Additionally, implementing a longitudinal program requires substantial and ongoing faculty, mentorship, and institutional support to ensure strong educational quality across multiple years.

## Challenges and Implementation Considerations

There are several challenges to implementing innovation-focused programs within a medical school curriculum. Notably, the highly structured and densely packed schedule of the medical school curriculum may leave limited room for additional courses or activities. However, the I&E program’s duration and content can be tailored around the existing schedule. Many medical schools allocate decided time for optional independent scholarly activities and research [6]. Portions of this time can be allotted to core classes in the I&E track or independent work. In the clinical years, students often have the option to take research electives, which can also provide additional time to engage with the I&E curriculum.

A phased implementation approach may help effectively introduce a health care I&E track, especially in institutions with limited resources. By initially offering short elective courses that require minimal infrastructure, institutions can assess student engagement and interest [12]. For example, during the 2024‐2025 academic year, we designed and led a three-week long elective course for first year medical students focused on teaching the principles of the design process and applying them to simple real-world problems. Fifteen (out of 120) first-year medical students registered for and then completed the course, reflecting high student interest. As faculty observe the value students place on I&E training, they may become more receptive to integrating these concepts into the broader curriculum. The positive outcomes from a pilot program can also build a compelling case for increased institutional support and may also help create a collaborative environment in which faculty and administration can collectively support the evolution of the medical curriculum.

Another challenge for the implementation of a health care I&E program is securing sustainable funding. One strategy is to pursue external funding sources dedicated to improving medical education. For example, the American Medical Association’s ChangeMedEd initiative offers Innovation Grants to support transformation educational projects. Another option is forming partnerships with health care organizations and technology companies. For example, collaborations with venture capital or medical device companies can provide students with projects to address real-world clinical needs. This offers students practical experience while also advancing the partners’ objectives. These partnerships also provide students with expertise and mentorship. In fact, the aforementioned CBID program successfully uses such a partnership model for many of the project opportunities offered to their students. In addition, at Johns Hopkins, pre- and postdoctoral training grants focused on health care I&E have provided support for some medical students and residents in pursuing yearlong Masters programs and projects.

## Future Directions and Expected Trends

We anticipate that interest in I&E education among medical students will continue to increase. First, growing student awareness of the importance of health care I&E is evident in our survey findings, where 43% of students considered innovation opportunities when choosing their medical school. Second, external forces, including health care system pressures, technological advancement, and changing care delivery models create increasing demand for physician innovators. Third, the increase in dual-degree and specialized programs reflects students’ desire to differentiate themselves for residency applications and career development [4,6]. Notably, 57% of the 28 identified programs were launched within the past four years, underscoring the recent surge in innovation-focused medical education.

Future studies should be conducted to further understand the role of I&E education in medical curricula. These studies should be larger and involve geographically diverse institutions across different tiers and program structures to validate the observations from our study. Additionally, longitudinal studies with larger cohorts that follow students for multiple years through medical training and into their careers will be important to capture the full effect of structured I&E training on career trajectories and professional contributions in health care.

## Conclusion

As medicine evolves at an ever-accelerating pace, it is increasingly important for the next generation of physicians to have a proper foundation in clinical problem solving and develop the design thinking principles and effective problem-solving skills that will allow them to integrate emerging technologies into their solutions and contribute to the creation new ones. Early exposure to health care I&E allows students to nurture both of these aspects of their academic development simultaneously, augmenting the quality of their overall training and promoting their ability to serve as physician leaders of health care I&E in the future. Our findings suggest substantial interest in I&E training among medical students at our institution, consistent with national trends, and we expect this trend to increase rather than diminish. Therefore, following in the footsteps of prior successfully implemented programs, we encourage all medical schools to investigate ways in which they can integrate I&E programs into their curriculum. These efforts will better prepare students to collaborate across disciplines and lead future innovation initiatives that will ultimately improve the care of the patients they will serve.

## Abbreviations

CBID: Center for Bioengineering Innovation and Design
HST: Health Sciences and Technology
I&E: Innovation and Entrepreneurship
